# The Eye as a Non-Invasive Window to the Microcirculation in Liver Cirrhosis: A Prospective Pilot Study

**DOI:** 10.3390/jcm9103332

**Published:** 2020-10-17

**Authors:** Fiona J Gifford, Francesca Moroni, Tariq E Farrah, Kirstie Hetherington, Tom J MacGillivray, Peter C Hayes, Neeraj Dhaun, Jonathan A Fallowfield

**Affiliations:** 1 Liver Unit, Royal Infirmary of Edinburgh, Edinburgh EH16 4SA, UK; fionagiff@googlemail.com (F.J.G.); francesca.moroni@nhs.net (F.M.); p.hayes@ed.ac.uk (P.C.H.); 2 Centre for Cardiovascular Science, University of Edinburgh, Edinburgh EH16 4TJ, UK; tariq.farrah@ed.ac.uk (T.E.F.); bean.dhaun@ed.ac.uk (N.D.); 3 Edinburgh Clinical Research Facility, University of Edinburgh, Edinburgh EH16 4SA, UK; kirstiehetherington@gmail.com; 4 Centre for Clinical Brain Sciences, University of Edinburgh, Edinburgh EH16 4SA, UK; T.J.MacGillivray@ed.ac.uk; 5 Centre for Inflammation Research, University of Edinburgh, Edinburgh EH16 4TJ, UK

**Keywords:** microcirculation, cirrhosis, optical coherence tomography, renal dysfunction

## Abstract

Microcirculatory dysfunction is associated with organ failure, poor response to vasoactive drugs and increased mortality in cirrhosis, but monitoring techniques are not established. We hypothesized that the chorioretinal structures of the eye could be visualized as a non-invasive proxy of the systemic microvasculature in cirrhosis and would correlate with renal dysfunction. Optical Coherence Tomography (OCT) was performed to image the retina in *n* = 55 cirrhosis patients being assessed for liver transplantation. OCT parameters were compared with established cohorts of age- and sex-matched healthy volunteers (HV) and patients with chronic kidney disease (CKD). Retinal thickness, macular volume and choroidal thickness were significantly reduced relative to HV and comparable to CKD patients (macular volume: HV vs. cirrhosis mean difference 0.44 mm^3^ (95% CI 0.26–0.61), *p* ≤ 0.0001). Reduced retinal thickness and macular volume correlated with renal dysfunction in cirrhosis (macular volume vs. MDRD-6 eGFR r = 0.40, *p* = 0.006). Retinal changes had resolved substantially 6 weeks following transplantation. There was an inverse association between choroidal thickness and circulating markers of endothelial dysfunction (endothelin-1 r = −0.49, *p* ≤ 0.001; von Willebrand factor r = −0.32, *p* ≤ 0.05). Retinal OCT may represent a non-invasive window to the microcirculation in cirrhosis and a dynamic measure of renal and endothelial dysfunction. Validation in different cirrhosis populations is now required.

## 1. Introduction

Decompensation and organ dysfunction in liver cirrhosis are characterised by systemic inflammation, regional microcirculatory alterations and profound systemic haemodynamic adaptations [1,2]. In patients with cirrhosis, splanchnic vasodilatation causes arterial ‘steal’ from the systemic circulation into the splanchnic bed [3], which decreases the effective blood volume and in turn triggers a variety of compensatory mechanisms. Marked changes occur in the renal circulation secondary to neurohormonal activation (renin-angiotensin-aldosterone system, sympathetic nervous system, vasopressin), a loss of renal autoregulation and an imbalance of intra-renal vasoconstrictors and vasodilators. Cardiac dysfunction (including cirrhotic or alcoholic cardiomyopathy) compounds circulatory derangements and kidney hypoperfusion. Accordingly, haemodynamic changes have been demonstrated in a range of extrahepatic vascular beds using modern vascular imaging techniques [4,5]. However, although the macrocirculation has been extensively characterised in cirrhosis, the microcirculation has been relatively understudied [6]. Emerging data suggest that, as in patients with severe sepsis [7], dysregulated systemic inflammation and microcirculatory alterations in different cirrhosis phenotypes may correlate with poor clinical outcomes [8]. Furthermore, despite normalisation of systemic haemodynamic variables in cirrhosis using fluids and vasoactive drugs, there is not necessarily a parallel improvement in microcirculatory perfusion and restoration of tissue oxygenation. This loss of haemodynamic coherence could explain the variability in response to terlipressin in patients with hepatorenal syndrome, illustrated by the drug’s heterogeneous effect on renal perfusion indices [9]. Assessment of the microcirculation could therefore play a potentially critical role in understanding the complex pathophysiology in an individual patient, monitoring of treatment interventions, and prognostication across different clinical states of cirrhosis.

Although there are no techniques to monitor the microcirculation in widespread clinical use, a number of modalities have recently been examined. In particular, novel handheld microscopes [10] have been used to visualise the sublingual microcirculation in critical illness (e.g., sepsis, high-risk surgery) [11] and also to study the effects of pharmacological therapies targeting the microcirculation [12]. The retinal vasculature is an established non-invasive proxy of systemic microvascular health. Optical coherence tomography (OCT) allows direct visualisation of chorioretinal microvascular structures. We recently used OCT to show that chorioretinal thinning in chronic kidney disease (CKD) is associated with lower eGFR and correlates with circulating markers of inflammation and endothelial function [13]. As renal (and other organ) dysfunction in decompensated cirrhosis is common, associated with a high mortality, and characterised by a systemic proinflammatory and pro-oxidant milieu, we hypothesised that OCT could be used to detect and monitor chorioretinal changes within the eye, providing a surrogate measure of the renal and extrahepatic microcirculations. Here we report an initial pilot study in a cohort of patients with liver cirrhosis undergoing assessment for liver transplantation and show significant chorioretinal alterations that correlated with renal function and markers of endothelial dysfunction. Furthermore, these OCT features were dynamic and resolved substantially following liver transplantation.

## 2. Experimental Section

### 2.1. Ethics

This observational study was conducted according to the ethical principles of the Declaration of Helsinki 2013 and following approval from the North West—Haydock Research Ethics Committee (REC Reference: 17/NW/0692) and the National Health Service (NHS) Lothian Research and Development department (Reference: 2017/0326). All patients gave written informed consent to participate in the study.

### 2.2. Participants

Consecutive male and female adult patients with liver cirrhosis admitted to the Edinburgh Transplant Centre (Royal Infirmary of Edinburgh, Edinburgh, UK) over a 6-month period were invited to join this study. Inclusion criteria were: male or female subjects over 18 years of age; patients with cirrhosis being assessed for liver transplantation; able to give informed consent and able to understand and willing to comply with the requirements of the study. Exclusion criteria were: lack of capacity to give informed consent; patients with acute liver failure being assessed for liver transplantation.

Permission was obtained to record the results of all investigations performed routinely as part of the NHS transplant assessment process. These data included: routine blood tests (full blood count, urea and electrolytes, liver function tests and coagulation); urinary sodium and creatinine clearance; anthropometric assessments. Estimated glomerular filtration rate (eGFR) was calculated using the Modification of Diet in Renal Disease-6 (MDRD-6) equation. The MDRD-6 equation has greater accuracy in patients with cirrhosis (compared to the traditional MDRD-4 equation) and the Organ Procurement and Transplantation Network consensus supports the use of MDRD-6 when assessing renal function in transplant assessment patients [14]. Results of additional tests including pulmonary function tests, cardio-pulmonary exercise testing, ECG and echocardiogram were recorded, but were not included in this analysis.

### 2.3. Study Visit

#### 2.3.1. Optical Coherence Tomography (OCT)

**Figure 1:**
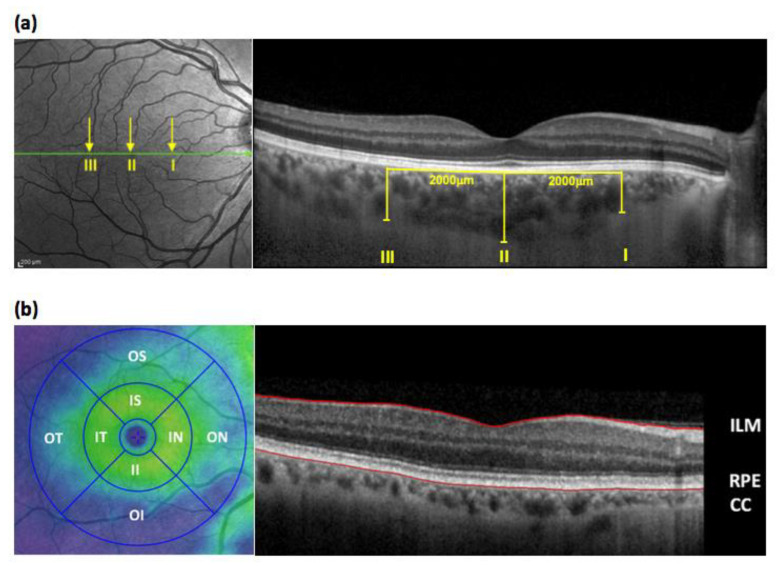
(**a**) The left panel is a representative en face retinal optical coherence tomography (OCT) image obtained using the Heidelberg SPECTRALIS^®^ OCT platform. The right panel is a cross sectional image taken at the level of the green line on the en face view. The line of the OCT scan passes through the fovea and optic disc. Choroidal thickness was measured manually at 3 locations on the macula using enhanced depth imaging technology (I: 2 mm nasal to the fovea, II = sub-foveal, III = 2 mm temporal to the fovea). (**b**) Sixty-one sequential horizontal line scans were performed covering the macular area. The retinal thickness of each area within the Early Treatment Diabetic Retinopathy Study (ETDRS) map (shown in the left panel) was automatically measured and then all areas combined to give the macular volume. The retinal layer is defined as the area between the internal limiting membrane (ILM) and the hypo-reflective line between the retinal pigment epithelium (RPE) and the choriocapillaries (CC) (depicted in the en face view of the macula shown in the right panel). The ETDRS map subdivides the macula, and retinal thickness was measured in eight zones (IS, inner-superior; IN, inner-nasal; II, inner-inferior; IT, inner-temporal; OS, outer-superior; ON, outer-nasal; OI, outer-inferior; OT, outer-temporal). All measurements were made by a trained technician who was blinded to all participant details.

Retinal assessment included retinal thickness, retinal nerve fiber layer (RNFL) thickness, macular volume and choroidal thickness as previously described [13], using the Heidelberg Spectralis OCT imaging platform that yields images with an axial or depth resolution of 3 μm/pixel and lateral resolution of 10 μm/pixel enabling identification of the retinal layers and choroid for quantification. The OCT imaging and analysis methodology is shown in Figure 1. Each procedure was performed under the same degree lighting (i.e., a dimmed room so as to avoid the need for pupillary dilatation) and took approximately 5–10 min to complete. Where possible, both eyes were scanned; however, images obtained from the right eye were preferentially used for analysis. In order to minimize bias, all OCT image analysis was performed by an expert assessor (Kirstie Hetherington) who was blinded to clinical status.

Imaging metrics (retinal thickness, RNFL thickness, macular volume, and choroidal thickness) in cirrhosis patients were compared with two pre-existing cohorts, of age- and sex-matched healthy volunteers (HV, *n* = 50) and patients with chronic kidney disease (CKD, *n* = 50), who had previously undergone OCT assessment on the same high-resolution Heidelberg SPECTRALIS^®^ platform.

#### 2.3.2. Sample Collection and Analysis

Blood was collected for routine serum biochemistry tests and plasma biomarker analysis. Validated ELISA kits were used to measure circulating levels of von Willebrand factor (Human von Willebrand Factor ELISA kit, #ab108918; Abcam, Cambridge, UK) and Endothelin-1 (Endothelin-1 Quantikine’ ELISA kit from, #DET100; R&D Systems, Abingdon, UK). A urine sample was collected for urinary protein to creatinine ratio (uPCR) and biomarker analysis.

### 2.4. Follow-Up 

Participants who were listed and received a liver transplant during the timeframe of the study were invited for a follow-up study visit at the Royal Infirmary of Edinburgh Clinical Research Facility, approximately 6 weeks after their transplant date. At this visit all study assessments were repeated. Morbidity data were collected for all transplanted patients including the warm ischaemic time, graft function at 6 weeks, the development of AKI or need for renal replacement therapy at the time of transplantation, length of Intensive Care Unit stay, and overall hospital stay.

### 2.5. Statistics

#### 2.5.1. Sample Size

This was a pilot study and, as such, the sample size was pragmatic, based upon the anticipated recruitment rate and study duration. Approximately 4–5 patients per week are admitted for liver transplant assessment, therefore based on a refusal rate of 50%, we anticipated recruitment of 54 patients over a 6-month period.

#### 2.5.2. Statistical analysis

Summary statistics (*n*, mean, standard deviation (SD), median, min, max) are presented for all recruited patients, and also for the subgroup who received a liver transplant during the period of the study to allow comparison. All data were assessed for normality, and log transformed if appropriate, before parametric tests were used. Two-tailed independent sample t-tests were used to compare continuous pre-transplant data according to AKI at transplantation, graft loss and survival (‘yes’ × ‘no’). Chi squared tests were used to examine relationships between liver disease severity scores and categorical outcomes. One-way analysis of variance (ANOVA) was used to compare continuous post-transplant outcomes according to liver disease severity (≥3 categories, e.g., Child-Pugh Class A/B/C). Pearson’s correlations were used to assess relationships between continuous pre-transplant and post-transplant data. A *p*-value < 0.05 was considered statistically significant. All statistics were calculated using IBM SPSS^®^ Statistics, version 24 (IBM, Armonk, NY, USA).

## 3. Results

### 3.1. Participant Disposition

A total of 55 patients with cirrhosis were recruited. Of these, two participants were too unwell to undergo OCT scanning, one participant was unwilling to attend, and three participants were unable to comply with the examination process. Results of the remaining 49 participants were used for analysis; 29 (59%) were male, mean age 58 ± 9 years and mean eGFR 100 ± 24 mL/min/1.73 m^2^. The mean Model for End-Stage Liver Disease (MELD) score was 14 (range 6–27) and the mean United Kingdom Model for End-Stage Liver Disease (UKELD) score was 53 (range 45–62). Seven (14%) participants had Child-Pugh (C-P) class A disease, 22 (45%) C-P class B, and 20 (41%) C-P class C. OCT imaging metrics in cirrhosis patients were compared with pre-existing cohorts of age- and sex-matched HV and CKD patients. Baseline patient characteristics are summarised in Table 1.

### 3.2. Chorioretinal Measurements in Cirrhosis 

#### 3.2.1. Chorioretinal Parameters in Cirrhosis, CKD and HV

**Figure 2:**
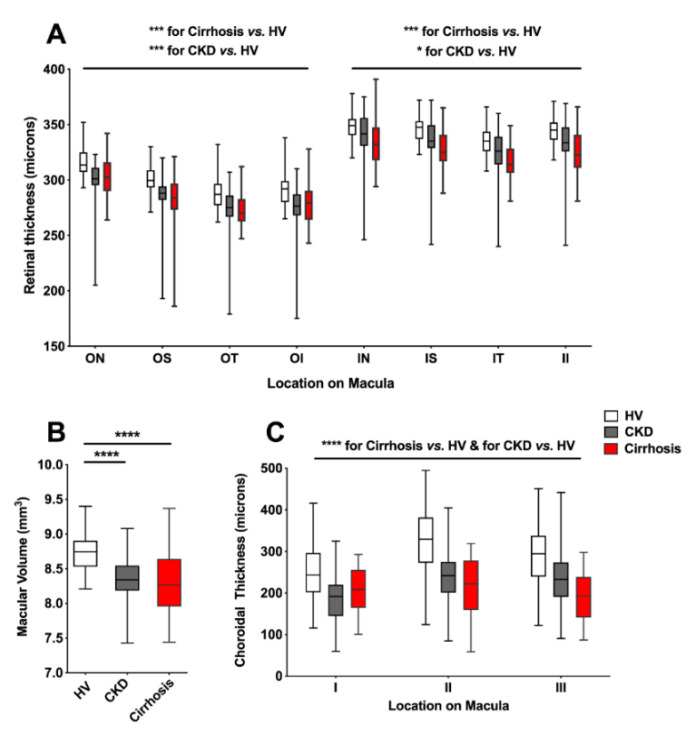
(**A**) Box and whiskers plot comparison of retinal thickness at each macular location between participants with cirrhosis, healthy volunteers (HV) and chronic kidney disease (CKD). ON; outer nasal, OS; outer superior, OT, outer temporal, OI; outer inferior, IN; inner nasal, IS; inner superior, IT; inner temporal, II; inner inferior. (**B**) Box and whiskers plot comparison of macular volume. (**C**) Box and whiskers plot comparison of choroidal thickness. All whiskers represent minimum to maximum. * *p* ≤ 0.05, *** *p* ≤ 0.001, **** *p* ≤ 0.0001.

Participants with cirrhosis had marked retinal thinning at all macular locations when compared to HV (F 82.3, *p* < 0.001) (Supplementary Table S1). These abnormalities were comparable to, or more severe than, those shown in CKD patients, despite marked disparity in eGFR (eGFR cirrhosis, mean ± SD; 100 ± 24 mL/min/1.73 m^2^, CKD: 37 ± 23 mL/min/1.73 m^2^ (Figure 2A). In keeping with a thinner retina, participants with cirrhosis had a significant reduction in macular volume (HV vs. cirrhosis mean difference 0.44 mm^3^, 95% CI 0.26–0.61, *p* < 0.0001) (Figure 2B; Supplementary Table S2). Moreover, in all three macular locations significant choroidal thinning was recorded (Figure 2C; Supplementary Table S3). This was most marked in locations II and III where the choroid was found to be ~30% thinner in cirrhosis relative to HV. No significant difference was found in either retinal thickness, macular volume or choroidal thickness between patients when grouped by aetiology of liver disease (alcohol related liver disease, chronic viral hepatitis, non-alcoholic fatty liver disease, primary biliary cholangitis, primary sclerosing cholangitis, or cryptogenic cirrhosis). 

#### 3.2.2. Correlation of Chorioretinal Parameters with Renal Function and Liver Disease Severity

**Figure 3:**
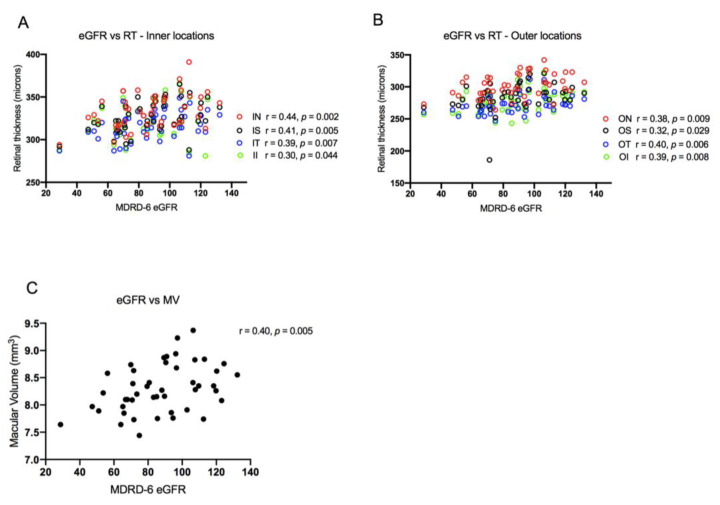
(**A**,**B**) Scatter plots of retinal thickness (RT) with estimated glomerular filtration rate (eGFR). (**C**) Scatter plot of macular volume (MV) and estimated glomerular filtration rate. Pearson correlation coefficients are shown (top right). IN, inner nasal; IS, inner superior; IT, inner temporal; II, inner inferior; ON, outer nasal; OS, outer superior; OT, outer temporal; OI, outer inferior. MDRD-6, Modification of Diet in Renal Disease-6 equation.

Retinal thickness and macular volume were shown to correlate significantly with (log-transformed) creatinine and eGFR. As renal function declined, so too did retinal thickness and macular volume (Figure 3). No significant association was found between MELD score and either retinal thickness or macular volume; however, the data suggested a non-significant trend towards lower retinal thickness and macular volume with increasing severity of liver disease, as defined by cirrhotic prognostic subgroup and variceal severity (Figures S2 and S3). Choroidal thickness did not correlate with renal function, MELD, or severity of liver disease.

#### 3.2.3. Alterations in Chorioretinal Parameters with Liver Transplantation

**Figure 4:**
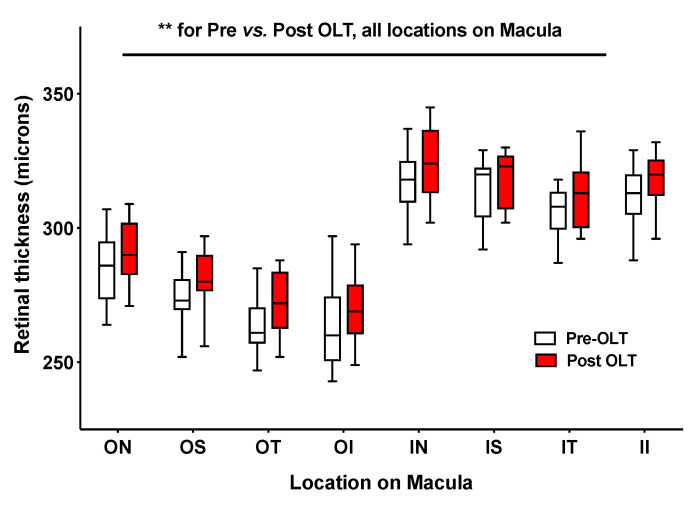
Box and whiskers plot of retinal thickness. Whiskers represent minimum and maximum. ** *p* ≤ 0.01; OLT, orthotopic liver transplant; ON, outer nasal, OS, outer superior; OT, outer temporal; OI, outer inferior; IN, inner nasal; IS, inner superior; IT, inner temporal; II, inner inferior.

A total of 14 participants underwent liver transplantation over the duration of the study and were invited back for repeat OCT. Three participants were lost to follow up (one participant died and two did not attend), and two were unable to comply with the examination process; therefore, comparison of chorioretinal parameters before and after transplantation was possible in nine participants. Retinal thickness (Figure 4; Supplementary Table S4) and macular volume measurements (Supplementary Table S5) had increased significantly 6 weeks after liver transplant (retinal thickness: F = 9.5, *p* = 0.003 (two-way mixed design ANOVA); macular volume pre-OLT (mean ± SD) 7.9 ± 0.3 mm^3^ vs. post-OLT 8.1 ± 0.3 mm^3^, *p* = 0.0007). No significant change was seen in choroidal thickness when re-measured after OLT. The data also suggested that choroidal thinning in location I, when measured at liver transplant assessment, may predict post-transplant acute kidney injury (AKI 170 ± 9 μm vs. no-AKI 231 ± 11 μm, *p* < 0.05) (Figure S1), although patient numbers were small. A similar pattern was seen at location II and III, however these data did not reach statistical significance.

#### 3.2.4. Chorioretinal Parameters and Markers of Inflammation and Endothelial Dysfunction

**Figure 5:**
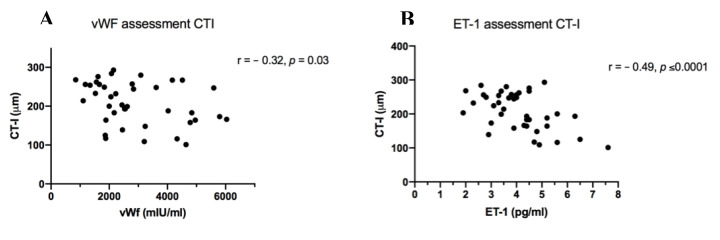
(**A**) Scatterplot of plasma von Willebrand factor (vWF) levels and choroidal thickness location I (CT-I). (**B**) Scatterplot of plasma endothelin-1 (ET-1) levels and CT-I. Pearson correlation coefficients are shown (top right).

Based on these data, further work was performed to explore the mechanistic roles of inflammation and endothelial dysfunction in mediating chorioretinal changes. Plasma von Willebrand factor (vWF; an endothelial activation marker) [15] and endothelin-1 (ET-1; an endogenous vasoconstrictor strongly linked with endothelial dysfunction) [16] were measured. Both vWF and ET-1 were markedly elevated before OLT, correlating significantly with severity of liver disease (MELD and variceal staging) and decreased substantially when rechecked 6 weeks after transplantation (Table 2). Moreover, there was a statistically significant, negative association between both plasma vWF and ET-1 level and choroidal thickness (Figure 5). No significant association was found between vWF or ET-1 and measures of retinal thickness and macular volume.

## 4. Discussion

Recent advances in multimodal retinal imaging devices enable non-invasive visualisation of the chorioretinal microvascular structures at high resolution. Examination of the microvasculature in this way has been used extensively in the research of both retinal and neurological disorders [17,18]. Moreover, retinal microvascular changes have been linked to increased cardiovascular risk [19,20], including the incidence of stroke [21] and coronary heart disease [22], suggesting that these abnormalities may reflect systemic microcirculatory dysfunction, and could represent an early, non-invasive technique to detect subclinical vascular pathology [22]. Cirrhosis is associated with widespread microcirculatory dysfunction and haemodynamic abnormalities. Correction of macrocirculatory derangement (fluid resuscitation and vasoactive drugs) does not always lead to microcirculatory improvement, and haemodynamic coherence is lost [23]. Such microcirculatory dysfunction is independently associated with adverse outcomes, even after normalisation of systemic haemodynamic parameters [24,25].

We have shown, to our knowledge for the first time, significant chorioretinal abnormalities in patients with cirrhosis of diverse aetiology, attending for liver transplant assessment. Compared to an age- and sex-matched cohort of healthy volunteers, participants with cirrhosis exhibited significant retinal thinning and reduced macular volume, with changes comparable to or more severe than those seen in CKD. Moreover, as in CKD, retinal thickness and macular volume were found to correlate significantly with eGFR. It is widely recognised that serum creatinine based estimating equations overestimate GFR by >20% in patients with cirrhosis [26]. It is possible that OCT scanning may represent a more effective indicator of renal risk (both acute kidney injury at transplantation and progressive renal dysfunction thereafter) when compared to serum creatinine or eGFR. Further work is required to understand the causality of these chorioretinal abnormalities and ascertain their ability to predict risk. Intriguingly, these chorioretinal abnormalities were dynamic, and reversed substantially following liver transplantation. Furthermore, the choroid (a dense microvascular network receiving >80% retinal blood flow) was ~30% thinner in cirrhosis compared with HV, representing significant vascular rarefaction [27]. Importantly, choroidal thinning was positively associated with markers of endothelial dysfunction (ET-1) and systemic inflammation (vWF). This is consistent with the theory that choroidal thinning may reflect systemic microvascular dysfunction. 

In a similar fashion, video microscopy (VM) has been used to facilitate in vivo visualisation of the sublingual microcirculation. Using this technique, Sakr et al. showed an association between the degree of microcirculatory dysfunction and progression to multiorgan failure and death in patients with septic shock [7]. Using the same technology, Sheikh et al. demonstrated a significant reduction in sublingual microvascular blood flow in patients with decompensated cirrhosis, compared to those with compensated disease [28]. Moreover, small but significant alterations in the sublingual microcirculation were shown in patients with cirrhosis compared to HV matched for age, sex, and cardiovascular risk factors [29]. However, a recent study using VM in combination with Near Infrared Spectroscopy did not show any association between peripheral microcirculatory parameters and the severity of liver disease [30].

A key observation in this pilot study was that chorioretinal abnormalities in cirrhosis patients resolved substantially following liver transplantation. Further work is required to validate our observations and to elucidate the cause(s) of these OCT changes in cirrhosis, such as the potential role of increased sympathetic tone. Indeed, while the choroidal circulation has autonomic innervation, the retinal circulation does not. Thus, the thinning of the outer retina and choroid would be consistent with increased sympathetic tone affecting the choroidal vasculature. We did not investigate measures of sympathetic activity (e.g., serum norepinephrine) in the current pilot study, but these would be an interesting area for future research in different cirrhosis settings, such as acute decompensation of cirrhosis. A limitation of this study was the small sub-group (*n* = 9) of patients transplanted within the study period. A more prolonged period of follow-up would increase the number of participants with chorioretinal data before and after OLT, improving the statistical power. Future studies could also use data linkage to explore whether these OCT metrics are predictive of renal, liver, and cardiovascular outcomes in cirrhosis populations. It is conceivable that chorioretinal microvascular changes may also represent a dynamic and accessible non-invasive response marker for guiding the use of vasoactive pharmacological agents in cirrhosis such as non-selective β-blockers for variceal prophylaxis or vasoconstrictors for hepatorenal syndrome. The recent development of portable OCT machines will permit evaluation in different clinical settings, including in patients who are too unwell to transfer to a research facility.

## Figures and Tables

**Table 1:** Participant Characteristics.

Participant Characteristics	Healthy Volunteer Cohort (*n* = 50)	Cirrhosis Cohort (*n* = 49)	CKD Cohort (*n* = 50)
***Demographics***			
**Age (years)**	50 ± 8	58 ± 9	53 ± 16
**Male sex, *n* (%)**	28 (56)	29 (59)	33 (66)
***Clinical measurements***			
**BMI (kg/m^2^)**	25.5 ± 4.3	27.3 ± 5.3	26.9 ± 5.0
**Systolic blood pressure (mmHg)**	129 ± 14	124.9 ± 17	134 ± 17
**Diastolic blood pressure (mmHg)**	81 ± 9	68.8 ± 9	78 ± 10
**Mean arterial pressure (mmHg)**	95 ± 16	87 ± 10	96 ± 10
**Serum creatinine (μmol/L)**	74 ± 11	70 ± 18	219 ± 126
***CKD stage***			
**1 (eGFR ≥ 90 + uPCR > 15)**	-	6	2
**2 (eGFR 60–89 + uPCR > 15)**	-	5	8
**3 (eGFR 30–59)**	-	2	20
**4 (eGFR 15–29)**	-	0	13
**5 (eGFR < 15)**	-	0	8
**Estimated GFR, mL/min/1.73 m^2^**	98 ± 13	100 ± 24	37 ± 23

Data shown as mean ± standard deviation unless stated. BMI, body mass index; CKD, chronic kidney disease; eGFR, estimated glomerular filtration rate; uPCR, urinary protein creatinine ratio.

**Table 2:** Serum levels of endothelin-1 (ET-1) and von Willebrand factor (vWF) pre and post orthotopic liver transplant (OLT).

	Pre OLT (*n* = 55)	Post OLT (*n* = 12)	*p*-Value
	mean ± SD	mean ± SD	
**vWF** (mIU/mL)	3047 ± 1330	1744 ± 637	0.006
**ET-1** (pg/mL)	3.9 ± 1.3	3.1 ± 0.6	0.06

Data shown as mean ± standard deviation (SD) with two-tailed paired *t*-test.

## Supplementary Materials

The following are available online at https://www.mdpi.com/2077-0383/9/10/3332/s1: Table S1. Two-way ANOVA results of retinal thickness in healthy volunteers (HV), chronic kidney disease (CKD) and cirrhosis patients; Table S2. Ordinary one-way ANOVA results of macular volume in healthy volunteers (HV), chronic kidney disease (CKD) and cirrhosis patients; Table S3. Two-way ANOVA results of choroidal thickness in healthy volunteers, chronic kidney disease and cirrhosis patients; Table S4. Results of retinal thickness before and after liver transplantation; Table S5. Paired t-test results of macular volume before and after liver transplantation; Figure S1. OCT variables and the development of acute kidney injury at liver transplant; Figure S2. Retinal thickness in different cirrhosis prognostic subgroups and variceal grades. Figure S3. Macular volume in different cirrhosis prognostic groups and variceal grades.
