# Supplementary material for: The Eye as a Non-Invasive Window to the Microcirculation in Liver Cirrhosis: A Prospective Pilot Study
Source: J Clin Med. 2020 Oct 17;9(10):3332. doi: 10.3390/jcm9103332 (PMC7603064; doi:10.3390/jcm9103332)
Supplement: Supplementary file 1 [file jcm-09-03332-s001.pdf]

Supporting Information

Table S1. Two-way ANOVA results of retinal thickness in healthy volunteers (HV), chronic kidney disease (CKD) and cirrhosis patients.

	Tukey's Multiple Comparisons Test	Predicted Mean Difference	95% CI of Difference	p-Value
ON	HV vs. CKD	17.1	8.1 to 26.0	≤0.0001
	HV vs. Cirrhosis	14.6	5.4 to 23.7	≤0.001
	CKD vs Cirrhosis	-2.5	-11.6 to 6.6	ns
OS	HV vs. CKD	16.1	7.2 to 25.1	≤0.0001
	HV vs. Cirrhosis	16.7	7.5 to 25.9	≤0.0001
	CKD vs Cirrhosis	0.56	-8.5 to 9.6	ns
OT	HV vs. CKD	15.2	6.2 to 24.2	≤0.001
	HV vs. Cirrhosis	15.8	6.7 to 25.0	≤0.001
	CKD vs Cirrhosis	0.65	-8.4 to 9/7	ns
OI	HV vs. CKD	18.0	9.0 to 26.9	≤0.0001
	HV vs. Cirrhosis	13.8	4.6 to 22.9	≤0.01
	CKD vs Cirrhosis	-4.2	-13.3 to 4.9	ns
IN	HV vs. CKD	10.1	1.2 to 19.1	≤0.05
	HV vs. Cirrhosis	15.2	6.1 to 24.4	≤0.001
	CKD vs Cirrhosis	5.1	-3.9 to 14.2	ns
IS	HV vs. CKD	19.2	1.3 to 19.2	≤0.05
	HV vs. Cirrhosis	18.2	0.1 to 27.4	≤0.0001
	CKD vs Cirrhosis	7.9	-1.1 to 17.0	ns
IT	HV vs. CKD	9.8	0.8 to 18.8	≤0.05
	HV vs. Cirrhosis	17.7	8.6 to 26.9	≤0.0001
	CKD vs Cirrhosis	7.9	01.1 to 17.0	ns
II	HV vs. CKD	13.1	4.1 to 22.1	≤0.01
	HV vs. Cirrhosis	19.0	9.9 to 28.2	≤0.0001
	CKD vs Cirrhosis	5.9	-3.1 to 15.0	ns

ns, not significant; CI, confidence intervals; ON; outer nasal, OS; outer superior, OT, outer temporal, OI; outer inferior, IN; inner nasal, IS; inner superior, IT; inner temporal, II; inner inferior; HV, healthy volunteers; CKD, chronic kidney disease.

Table S2. Ordinary one-way ANOVA results of macular volume in healthy volunteers (HV), chronic kidney disease (CKD) and cirrhosis patients.

Tukey's Multiple Comparisons Test	Mean Difference (mm^3)	95% CI of Difference	*p*-Value
HV vs. CKD	0.39	0.22 to 0.56	≤0.0001
HV vs. Cirrhosis	0.44	0.26 to 0.61	≤0.0001
CKD vs. Cirrhosis	0.05	-0.12 to 0.22	ns

CI, confidence intervals; HV, healthy volunteers; CKD, chronic kidney disease; ns, not significant

Table S3. Two-way ANOVA results of choroidal thickness in healthy volunteers, chronic kidney disease and cirrhosis patients.

	Tukey's Multiple Comparisons Test	Predicted Mean Difference (μm)	95% CI of Difference	p-Value
I	HV vs. CKD	64.9	32.4 to 97.3	<0.0001
	HV vs. Cirrhosis	40.9	7.1 to 74.7	<0.05
	CKD vs Cirrhosis	-23.9	-57.9 to 10.1	ns
II	HV vs. CKD	86.5	54.1 to 119.0	<0.0001
	HV vs. Cirrhosis	110.9	77.1 to 144.7	<0.0001
	CKD vs Cirrhosis	24.4	-9.6 to 58.3	ns
III	HV vs. CKD	59.5	27.0 to 91.9	<0.0001

HV vs. Cirrhosis	100.3	66.5 to 134.1	<0.0001
CKD vs Cirrhosis	40.8	6.8 to 74.8	<0.05

CI, confidence intervals; HV, healthy volunteers; CKD, chronic kidney disease; ns, not significant

Table S4. Results of retinal thickness before and after liver transplantation.

Retinal thickness (μm)		Study Participant								
		1	2	3	4	5	6	7	8	9
ON	Pre	286	297	264	290	293	274	273	307	279
	Post	291	298	271	290	306	283	285	309	282
OS	Pre	271	280	252	280	282	273	268	291	271
	Post	274	279	256	291	289	280	282	297	280
OT	Pre	254	272	247	261	269	260	260	285	263
	Post	261	274	252	267	287	272	-	288	272
OI	Pre	258	278	244	263	271	243	257	297	260
	Post	274	284	249	269	271	268	253	294	268
IN	Pre	329	337	306	317	321	318	294	320	313
	Post	334	339	313	313	345	302	327	324	322
IS	Pre	320	329	298	322	317	322	292	323	310
	Post	323	328	305	309	326	302	330	324	320
IT	Pre	310	318	295	308	307	313	287	314	304
	Post	312	319	298	302	336	296	323	315	313
II	Pre	323	329	299	313	314	311	288	317	311
	Post	326	332	304	320	320	296	325	322	320

ON; outer nasal, OS; outer superior, OT, outer temporal, OI; outer inferior, IN; inner nasal, IS; inner superior, IT; inner temporal, II; inner inferior

Table S5. Paired *t*-test results of macular volume before and after liver transplantation.

Pre OLT (mean ±SD, mm^3)	Post OLT MV (mean ±SD, mm^3)	*t*-value	Degrees of Freedom	*p*-Value
7.9 ±0.3	8.1 ±0.3	5.4	8	0.0007

Two-tailed *p*-value; OLT, orthotopic liver transplant.

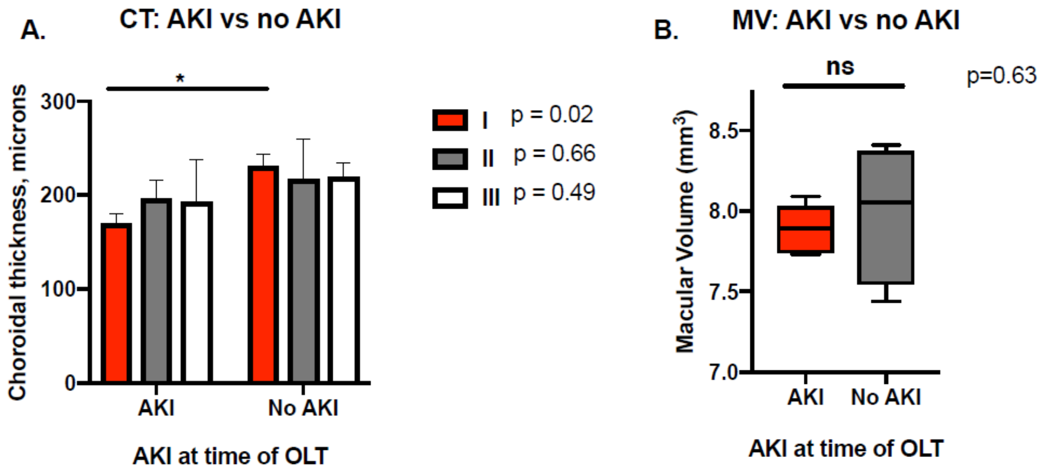

Figure S1. OCT variables and the development of acute kidney injury at liver transplant. CT, choroidal thickness; MV, macular volume; AKI, acute kidney injury; OLT, orthotopic liver transplantation.

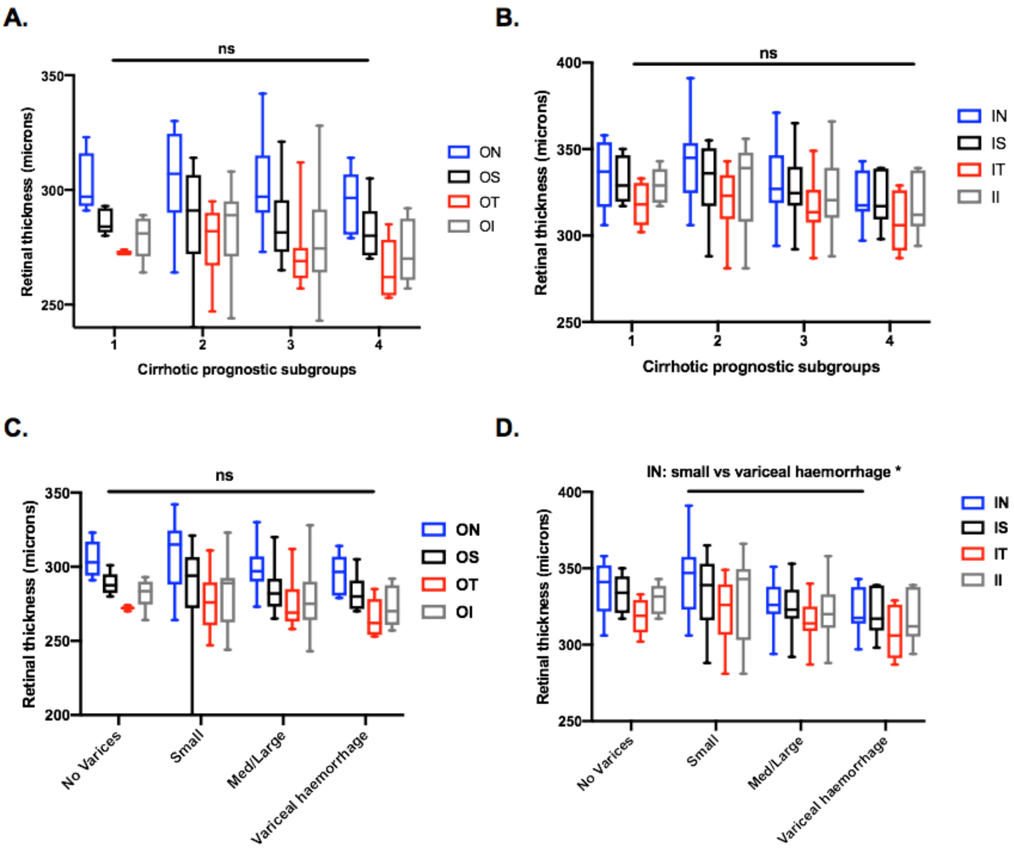

Figure S2. Retinal thickness in different cirrhosis prognostic subgroups and variceal grades. Box and whisker plots - middle line, boxes and whiskers represent median, interquartile range, and minimum and maximum, respectively. (**A**) Cirrhosis prognostic subgroup versus retinal thickness - outer locations. (**B**) Cirrhosis prognostic subgroup versus retinal thickness - inner locations. (**C**) Variceal grade and retinal thickness – outer locations. (**D**) Variceal grade vs retinal thickness – inner locations. RT, retinal thickness; ON, outer nasal; OS, outer superior; OT, outer temporal; OI, outer inferior; IN, inner nasal; IS, inner superior; IT, inner temporal; II, inner inferior. ns, $p > 0.05$; *, $p \leq 0.05$.

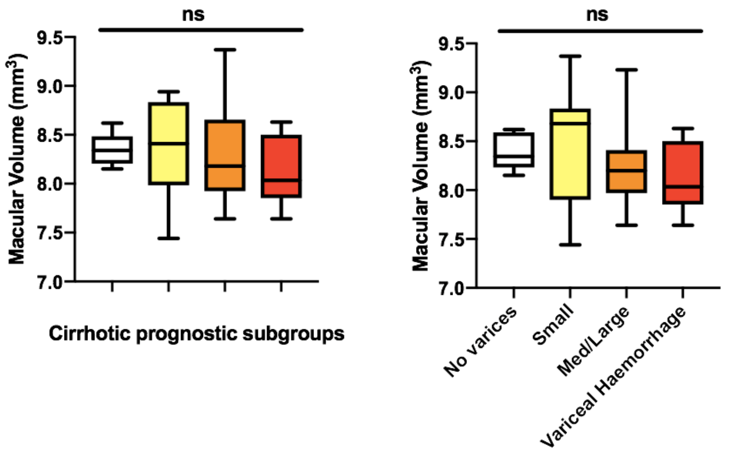

Figure S3. Macular volume in different cirrhosis prognostic groups and variceal grades. Box and whisker plots – middle line, boxes and whiskers represent median, interquartile range, and minimum and maximum, respectively. (**A**) Cirrhosis prognostic subgroup versus macular volume, (**B**) Variceal grades versus macular volume. ns, $p > 0.05$.
